# Recovering from a broken heart

**DOI:** 10.7554/eLife.87550

**Published:** 2023-04-18

**Authors:** Alison M Bell

**Affiliations:** 1 Department of Evolution, Ecology and Behavior, University of Illinois Urbana-Champaign United States

**Keywords:** prairie vole, nucleus accumbens, loss, Other

## Abstract

A molecular signature found in the brains of monogamous prairie voles begins to decay after prolonged separation from their partner.

**Related research article** Sadino JM, Bradeen XG, Kelly CJ, Brusman LE, Walker DM, Donaldson ZR. 2023. Prolonged partner separation erodes nucleus accumbens transcriptional signatures of pair bonding in male prairie voles. *eLife* **12**:e80517. doi: 10.7554/eLife.80517.

Despite the best efforts of my hole-digging dog, my backyard is home to rodents who are in love. Furry prairie voles spend lots of time huddled underground, usually in pairs that work together to rear their pups. If the couple is separated, even for as long as two weeks, they still remain bonded and will reunite as tight as they were before (Getz et al., 1981). These humble rodents are famous for their monogamous behavior, and have been the subject of many eye-catching headlines (Tucker, 2014; Armitage, 2015). Part of their appeal is the possibility to understand the biological basis of love, devotion, and life-long attachment – topics that seem more suited to humanities than biology departments.

Most scientific research on prairie voles has focused on how monogamous pairs are formed, particularly the role of oxytocin, the famous ‘love hormone’ (Insel and Shapiro, 1992). However, less is known about what happens when these bonds get broken; what happens when one member of the pair outlives the other? Previous behavioral studies have shown that voles exhibit signs of distress when they are separated from their partner, but eventually adapt to this loss and seek out a new connection (Harbert et al., 2020). Now, in eLife, Zoe Donaldson and colleagues from the University of Colorado Boulder and Oregon Health and Science University – including Julie Sadino as first author – report what happens to voles as they recover from a broken heart (Sadino et al., 2023).

Sadino et al. studied male voles in same-sex and opposite sex pairs that had been housed together for two weeks and then separated for 48 hours or four weeks. Using a technique called vTRAP (short for translating ribosome affinity purification), they investigated which genes are expressed in the nucleus accumbens, a part of the brain that is engaged during pair formation in mammals, including humans (Walum and Young, 2018). The pattern of genes expressed (also known as the transcriptional signature) was measured before and after voles were separated, along with their behavior to see if these two factors are coordinated together. The female voles were also prevented from becoming pregnant to control for the potentially confounding influence of co-parenting together.

The experiments revealed that male voles stably express hundreds of genes in their nucleus accumbens when living with a partner, which remained unchanged even after the pair were separated for a few days. However, while the male voles continued to prefer the company of their partner even after long periods apart, the transcriptional signature in the nucleus accumbens started to decay as the voles spent more time away from each other.

These findings suggest that genes in the brains of the voles start to alter their expression before the voles lose preference for their old mate. This means that although voles may behaviorally resume their relationship after long periods of separation, elements of their transcriptome may be less optimistic that this reunion will happen. It is tempting to speculate that the genes that begin to change expression may somehow be involved in recovering from the loss of a partner.

Furthermore, while behavioral and genomic changes in the brain occurring over different timescales is not an entirely new finding (White et al., 2002), the results of Sadino et al. do offer some clues as to how this may work at a mechanistic level. For example, genes that were upregulated in bonded males, but downregulated after a few weeks of separation, follow a pattern that suggests they may have a role in coping with loss. In contrast, another set of genes, which was initially downregulated and then upregulated after weeks of separation, may be involved in helping to prime the vole to form a new bond.

Understanding the neural basis of complex social behaviors is a hard problem. However, the work by Sadino et al. – which uses a savvy experimental design to connect genes, the brain and animal behavior – shows how it can be done. Moreover, and somewhat surprisingly, other researchers recently reported that prairie voles do not require a receptor for oxytocin in order to form social attachments (Berendzen et al., 2023). It seems that there are actually many players coordinating this complex social behavior.

An immediate task for future work is to look at brain areas other than the nucleus accumbens, which is closely associated with reward. While pair bonding is rewarding, this may be independent from the cognitive aspects of separation, such as the memory of a lost companion or the willingness to find a new partner. Another priority is to explicitly connect the transcriptional signatures identified with the formation or degradation of the pair bond using functional tests, a challenge the scientific community studying these furry lovers is no doubt going after next.
